# Influence of Different Surgical Timing after Percutaneous Kyphoplasty for Osteoporotic Vertebral Compression Fractures: A Retrospective Study

**DOI:** 10.1155/2022/7500716

**Published:** 2022-06-26

**Authors:** Hao Liu, Wenhao Wang, Yixue Huang, Xiayu Hu, Xuefeng Li, Huilin Yang

**Affiliations:** ^1^Department of Orthopaedics, The First Afﬁliated Hospital of Soochow University, Suzhou 215006, China; ^2^Orthopaedic Institute, Medical College, Soochow University, Suzhou 215000, China

## Abstract

**Background:**

A large number of people suffer from osteoporotic vertebral compression fractures (OVCFs) worldwide. Percutaneous kyphoplasty (PKP), considered a minimally invasive surgery, has been widely used to treat OVCFs and achieves satisfactory outcomes. However, the surgical timing of PKP is still under discussion.

**Methods:**

A total of 149 patients were enrolled in the study and were divided into 3 groups according to different surgical timing. Group A (*n* = 52) included patients who required emergency surgery. Group B (*n* = 50) included patients who required surgery around a week after injury. Group C (*n* = 47) included patients who required surgery a month or more after injury. Characteristics of patients and radiological images were recorded. The Visual Analog Scale (VAS) scores and the Oswestry Disability Index (ODI) scores were analyzed before, 1 day, 1 month, and 6 months after surgery. In addition, compression rates of anterior vertebral height (AVH) were calculated and the kyphosis Cobb angle was measured before and after surgery.

**Results:**

There was a significant difference in the VAS and ODI scores between the three groups at 1 day, 1 month, and 6 months after PKP. The VAS and ODI scores of Group C were higher than those of Groups A and B. The AVH compression rates of Group C were significantly higher than those of Groups A and B postoperatively 1 day, 1 month, and 6 months. The kyphosis Cobb angles in Group C was significantly larger than those in Groups A and B at 1 day and 1 month after PKP.

**Conclusions:**

Emergency PKP showed more advantages in both clinical and radiological outcomes. We recommend early PKP for the treatment of OVCFs.

## 1. Introduction

In 1885, European pathologists first proposed the term “osteoporosis”. After the 1960s, many experts conducted in-depth research on the etiology, predisposing factors, clinical diagnosis, and prevention of osteoporosis. For now, primary osteoporosis is known as a systematic skeletal disease which is characterized by decreased bone mass, destruction of bone microstructure, and increased bone fragility, caused by a combination of genetic and environmental factors. Over 200 million people worldwide suffer from osteoporosis [1]. Postmenopausal women and elderly men are more likely to be tortured by osteoporosis and its complications. China has gradually entered an aging society, and the harm of osteoporosis to society is becoming severe. According to recent data, the number of patients with osteoporosis in China is about 60 to 80 million, and the incidence of osteoporosis in women is 6 to 8 times higher than that in men. Complications of osteoporosis, such as osteoporotic vertebral compression fractures (OVCFs), reduce patients' quality of life, shorten life expectancy, and increase medical expenditures [2]. This not only causes physical and psychological harm to patients, but also increases the burden on families and society [3]. Under such a severe situation, how to better solve the complications of osteoporosis has become one of the focuses.

Percutaneous kyphoplasty (PKP) for OVCFs has been recognized as an effective and safe minimally invasive surgery. In 1987, Galibert and Deramond for the first time applied percutaneous vertebroplasty (PVP) to the treatment of C2 hemangioma, which has the pros of rapid pain relief, immediate fixation, and early mobilization [4]. However, one of the disadvantages of PVP is the high cement leakage rate, about 30% to 67%. In 1998, the Food and Drug Administration (FDA) approved the clinical trial of the balloon. In 2001, Lieberman et al. for the first time reported PKP for treating OVCFs [5]. At this point, this surgery announced that PKP boarded the stage of treating OVCFs. Compared to traditional surgery, PKP has the advantages of less trauma, short operation time, quick pain relief, and short hospitalization period. Compared to PVP, many scholars hold the view that PKP has a lower bone cement leakage rate, which improves the safety of the minimally invasive surgery.

With continuous research on PKP in clinical practice, many problems, such as the volume of bone cement and the piercing angle, have been gradually solved. However, some issues are still under debate. For example, scholars still have controversy over the timing of PKP for treating OVCFs. Different surgery timing may result in different clinical efficacy. Here, we authors put forward the following question: early and delayed PKP, which is more advantageous?

## 2. Methods

### 2.1. Objectives

Patients with single-level OVCFs who were admitted to the orthopedics department of our hospital from October 2015 to October 2020 were enrolled in this study. The inclusion criteria were as follows: (1) single-level thoracic or lumbar osteoporotic compression fracture; (2) caused by mild violence such as sprains and falls; (3) without neurological deficit; and (4) with follow-up data for at least 6 months. The exclusion criteria were as follows: (1) burst fracture; (2) under the age of 50; (3) caused by severe violence such as car accident; (4) with symptoms of nerve compression; and (5) pathological fractures caused by tumor, infection, and other diseases.

A total of 149 patients were enrolled in the study. Patients underwent PKP under general anesthesia. The bilateral approach was adopted and bone cement was used during each PKP. There were 3 groups (A, B, and C) according to different surgery timing. Group A (*n* = 52) required emergency surgery (≤24 hours). Group B (*n* = 50) required surgery around a week (6–8 days) after injury. Group C (*n* = 47) required surgery after a month or more (≥30 days) after injury. All patients' data and images were obtained from the electronic medical record management system of the hospital. This study was approved by the ethics committee of the hospital.

### 2.2. Surgical Procedure

Patients underwent PKP under general anesthesia. Each patient was prone positioned. C-arm fluoroscopy was used in the perioperative period to locate the fractured vertebra and confirm bone cement dispersion. The bilateral approach was adopted for each patient. Small incisions were made on the skin. Afterward, needles were inserted into the fractured vertebra under the guidance of C-arm fluoroscopy. Then, needles were withdrawn, guide pins were inserted, and working tubes were placed. Fine drills were used to drill holes and the balloons were extended. Bone cement (KYPHON®Xpede^TM^) was injected into the fractured vertebra. When the cement diffusion reached the edge of the vertebra, the injection was stopped. The same group of doctors performed PKP for each patient.

### 2.3. Evaluation

Patient characteristics were recorded and assessed after they were admitted to our department, including demographic data and perioperative parameters. X-rays were taken before and 1 day after surgery. CT (computer tomography) and MRI (magnetic resonance imaging) were also taken before PKP. Visual Analog Scale (VAS) scores and the Oswestry Disability Index (ODI) scores were recorded to assess the rehabilitation of patients before, 1 day, 1 month, and 6 months after surgery. The VAS aimed to evaluate pain and the ODI aimed to evaluate functional disorders. Anterior vertebra heights (AVH) were measured by lateral radiography before, 1 day, 1 month, and 6 months after surgery. The compression rates were calculated by the following equation: Compression rate of AVH (%) = 1–2^*∗*^H1/ (H2 + H3), where H1, H2, and H3 are the anterior heights of the fractured vertebra, the vertebra above the fractured vertebra, and the vertebra below the fractured vertebra, respectively. The Kyphosis Cobb angle was also measured before and after PKP. We measured the angle between the superior endplate of the vertebra above the fractured vertebra and the inferior endplate of the vertebra below the fractured vertebra after drawing lines and perpendiculars.

### 2.4. Statistical Analysis

The SPSS 23.0 statistical software was used for data analysis. ANOVA was used to compare the data of three groups. After ANONA, LSD was adopted to compare data of different two groups (Groups A and B, A and C, and B and C). The chi-squared test was used to compare variables of categorical data. Data were presented as mean ± standard deviation. GraphPad Prism was used to draw histograms. *P* < 0.05 was considered statistically significant.

## 3. Results

### 3.1. Characteristics of the Patients

The demographic data and perioperative parameters of the three groups are shown in Table 1. It was observed that the number of female patients was more than that of male patients in each group. Also, lumbar fractures were more than thoracic fractures. There was no significant difference in BMI, operative time, blood loss, and cement volume between the three groups. However, the hospital stay of Group C was significantly longer than that of Groups A and Group B (6.57 ± 1.63, 5.71 ± 1.66, and 5.74 ± 1.32, respectively, *P* < 0.01).

### 3.2. Clinical Outcomes

**Figure 1:**
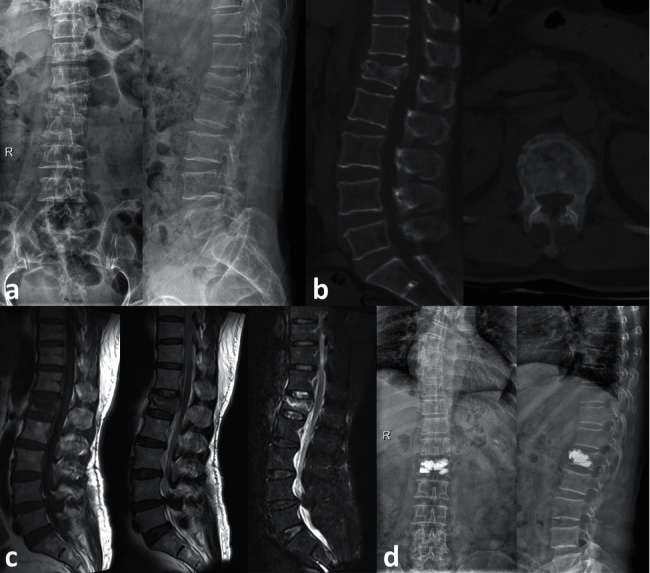
A 62-year-old female with an L1 OVCF who underwent PKP. (a) X-rays before PKP. The compression fracture of L1 can be observed. (b) CT before PKP. (c) MRI before PKP, including T1, T2, and STIR. (d) X-rays 1 day after surgery.

**Figure 2:**
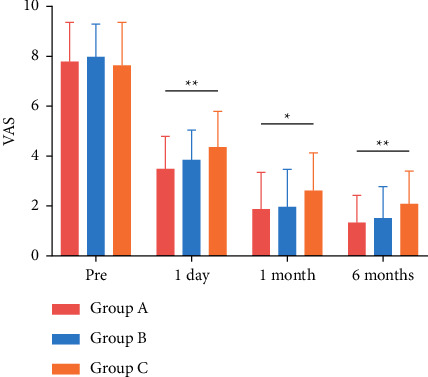
Comparison of Visual Analog Scale (VAS) scores. ^*∗*^*P* value indicates statistical significance between the three groups (*P* < 0.05). ^*∗∗*^*P* value indicates statistical significance between three groups (*P* < 0.01).

**Figure 3:**
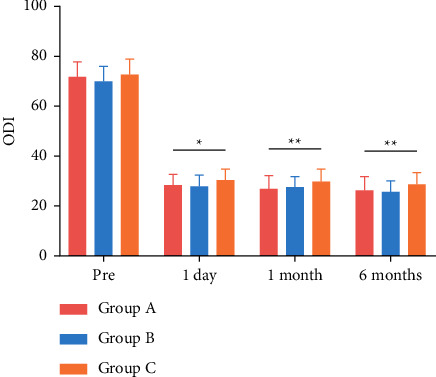
Comparison of the Oswestry Disability Index (ODI) scores. ^*∗*^*P* value indicates statistical significance between three groups (*P* < 0.05). ^*∗∗*^*P* value indicates statistical significance between three groups (*P* < 0.01).

X-rays before and 1 day after surgery, and CT and MRI before PKP are all shown in Figure 1. The VAS scores of the three groups are shown in Figure 2, and the ODI scores are presented in Figure 3. According to the results, VAS and ODI scores of the three groups at 1 day, 1 month, and 6 months after surgery all decreased significantly compared with those before surgery (*P* < 0.05). In addition, there was a significant difference in the VAS and ODI scores between the three groups at 1 day, 1 month, and 6 months after PKP (*P* < 0.05). The VAS and ODI scores of Group C were higher than those of Groups A and B.

### 3.3. Radiographic Outcomes

The compression rates of AVH are shown in Table 2. Compared with the preoperative rates, the compression rates of AVH were significantly improved in the three groups at 1 day, 1 month, and 6 months after PKP (*P* < 0.05, *P* < 0.05). The AVH compression rates of Group C were significantly higher than those of Groups A and B at 1 day, 1 month, and 6 months after surgery (*P* < 0.05). However, there was no significant difference in the compression rates of AVH between Groups A and B (*P* > 0.05).

The kyphosis Cobb angle of three groups is shown in Table 3. Compared with preoperative results, the kyphosis Cobb angle all decreased significantly in three groups at 1 day, 1 month, and 6 months after surgery (*P* < 0.05). Additionally, the kyphosis Cobb angle of Group C was significantly larger than that in Groups A and B at 1 day and 1 month after PKP (*P* < 0.05). There were no significant differences in the kyphosis Cobb angle between Groups A and B (*P* > 0.05).

## 4. Discussion

OVCF is a common disease that causes acute or chronic pain, spinal deformity, and severe dysfunction in the elderly. The disease greatly reduces the quality of patients' lives. About 1,700,000 vertebral compression fractures occur each year in the United States and Europe. Unfortunately, the number is still growing in the coming decades as the population ages, bringing a huge medical and economic burden for society [3,6,7]. Osteoporotic fractures often occur with mild violence, weight lifting, or even without any trigger. It is reported that only about one-third of patients with fresh OVCFs receive treatments, suggesting that the pain is not severe and patients usually endure the pain [8]. The pain caused by fresh vertebral fractures usually lasts for 4–8 weeks, and percussion pain is obvious at the fractured vertebra [9].

Conservative treatments, including bed rest, nonsteroidal anti-inflammatory drugs, and antiosteoporosis therapy, are widely used for patients with less than 20° kyphosis and 25% vertebral height loss [10]. Long-term bed rest for patients with OVCFs accelerates the progress of bone loss and increases the risk of pneumonia, ulcers, and vein thrombosis. Such complications lead to approximately 50% of fracture-related deaths [11]. In addition, a single-segment OVCF increases the risk of new vertebral fractures fivefold in the first year, and two or more segment fractures increase the risk up to 12-fold [12,13]. Therefore, surgery is particularly recommended for patients with progressive symptoms and kyphosis [14]. PVP and PKP are regarded as mature minimally invasive surgery for treating OVCFs with advantages of less blood loss, less trauma, immediate pain relief, and early mobilization [15]. Recent data showed that PKP could significantly relieve pain, improve function, and quality of life [16]. PKP is also considered a better intervention for decreasing the risk of subsequent vertebral fracture and refracture compared with conservative treatments [17]. However, in some cases with poor conditions or economic burden, conservative treatments should be adopted first.

Our study mainly focused on patients with OVCFs over the age of 50. In most countries, the age of menopause for women is around 50. People over the age of 50 especially postmenopausal women are more likely to suffer from OVCFs. In addition, patients under the age of 50 may tolerate open surgery, and internal fixation is also recommended to achieve stability. Therefore, we excluded patients under the age of 50 so as to make cases more typical and suitable for the treatment of PKP. In terms of clinical efficacy, we found that early surgery for patients significantly improved VAS and ODI scores. Better clinical outcomes were obtained after emergency surgery. Takahashi et al. showed that lower VAS scores were recorded in the early surgery group (<2 months) than in the delayed surgery group (>2 months) [18]. However, some studies have pointed out that the pain can be significantly relieved after both early and delayed surgery [18]. Since bone cement can act as a riveter and reduce nerve sensitivity, pain can be significantly reduced. It was proposed that the heat and chemical stimulation of the bone cement destroyed the terminal nerve in the fractured vertebra and thus relieved pain [19]. In addition, postoperative treatments of osteoporosis may be helpful for pain relief as well. Our results showed that there was no significant difference in the restoration of AVH and the kyphosis Cobb angle of the fractured vertebra between Group A and Group B. However, the radiographic outcomes of the two groups were all significantly better than the delayed surgery group. Since expansion with high pressure could result in balloon rupture, expansion with normal pressure should be adopted in the operation. It was indicated that osteotylus formation had occurred before the delayed surgery, which made it difficult to restore the physiological height of the vertebra by balloon expansion with normal pressure. As the osteotylus formation increased, the loss of vertebra height might become irreversible. In postoperative follow-up, it was not surprising that the clinical outcomes of the delayed surgery group were worse and the hospital stay was much longer than the early surgery group.

Early PKP can help stabilize the vertebra, improve the physiological curvature, and relieve muscle spasms. In the healing period, the in-growth of granulation tissue and the release of inflammatory factors may lead to an inflammatory response and eventually result in poor pain relief. However, it was reported that performing PKP 3 weeks after injury could lead to a higher rate of cement leakage, possibly due to cortical fissures [14]. In the subacute phase, the partial or complete fibrous tissue healing process prevents cement leakage from happening. The experienced surgeons could adjust the “state” of cement, injection time, and pressure to avoid cement leakage with the assistance of C-arm fluoroscopy. In addition, it has been put forward in another study that the damage of the anterior column could increase the pressure on adjacent vertebrae, and early intervention might help improve the stability of the spine and avoid fractures of the adjacent vertebrae [20]. Therefore, performing PKP in the subacute phase may be a better choice. Contrary to early PKP, delayed surgery may bring some disadvantages. The patient's center of gravity is shifted forward due to kyphosis, which results in a higher buckling moment around the kyphosis apex [9,21]. It may cause aggravated pain and an increased risk of fractures. The delayed surgery also leads to longer use of medication, which often comes with side effects such as kidney failure and addiction [22]. Although our study suggested that early PKP had more advantages, PKP is still an irreplaceable option for symptomatic long-term OVCFs. It was shown that the vertebra height could be restored in approximately 20% of patients with long-term OVCFs [23]. It was reported that PKP was able to stop the progress of kyphosis after OVCFs, but no evidence was shown that PKP could effectively improve the long-term sagittal alignment [24].

There are several limitations in our study. First, this is a retrospective study. There were limitations due to the study design compared with the prospective study. Second, although we measured the radiographic outcomes on X-rays, the accuracy is still not enough. Third, our study only included 149 patients. Long-term follow-up is needed to better analyze the data. Randomized controlled trials with larger sample sizes are also needed in future.

## 5. Conclusions

In summary, we believe that emergency surgery has faster recovery and better radiological outcomes. However, it is necessary to ensure the result of blood tests, electrocardiograms, and other examinations before emergency surgery. There was no significant difference in the recovery for patients who underwent PKP 1 week after injury. However, persistent pain and acute bone loss greatly reduce the quality of life. Performing PKP 1 month or more after injury achieved the worst clinical outcomes among the three groups. Due to osteophyte formation and kyphosis, both the height and the kyphosis Cobb angle of the fractured vertebra were difficult to restore. Therefore, we believe that early PKP is more advantageous and it is strongly recommended for the treatment of OVCFs.

## Figures and Tables

**Table 1:** Characteristics of patients.

	Group A	Group B	Group C	*P* value
Cases (*n*)	52	50	47	
Age (years)	70.48 ± 9.64	68.42 ± 10.65	67.92 ± 8.77	0.377
Gender (*n*, males/females)	10/42	9/41	11/36	0.786
Level (*n*, thoracic/lumbar)	17/35	16/34	18/29	0.775
BMI (kg/m^2^)	24.88 ± 3.50	26.30 ± 3.36	25.37 ± 2.83	0.085
Operative time (min)	48.02 ± 7.62	49.10 ± 9.90	49.77 ± 10.11	0.636
Blood loss (ml)	9.42 ± 4.04	9.60 ± 4.39	10.32 ± 5.46	0.603
Cement volume (ml)	6.24 ± 1.32	6.15 ± 1.18	6.34 ± 1.20	0.751
Hospital stay (days)	5.71 ± 1.66	5.74 ± 1.32	6.57 ± 1.63	0.009^*∗∗*^

BMI: body mass index. ^*∗∗*^*P* value indicates statistical significance (*P* < 0.01).

**Table 2:** Comparison of compression rates of AVH (%) between the three groups.

Group	Cases	Preoperation	1 day after operation	1 month after operation	6 months after operation
A	52	15.25 ± 10.42	10.62 ± 4.69	10.15 ± 5.42	10.59 ± 4.84
B	50	17.24 ± 7.54	10.28 ± 5.58	9.89 ± 4.54	10.38 ± 3.50
C	47	14.34 ± 9.15	12.91 ± 5.85	12.49 ± 4.78	12.60 ± 4.42
*P* value		0.278	0.036^*∗*^	0.020^*∗*^	0.022^*∗*^

^*∗*^ *P* value indicates statistical significance (*P* < 0.05).

**Table 3:** Comparison of the kyphosis Cobb angle (degrees) between the three groups.

Group	Cases	Preoperation	1 day after operation	1 month after operation	6 months after operation
A	52	23.75 ± 2.11	15.87 ± 2.30	16.19 ± 2.79	16.62 ± 2.58
B	50	24.30 ± 2.59	15.50 ± 2.16	16.08 ± 2.86	16.52 ± 2.85
C	47	24.09 ± 2.83	16.87 ± 2.20	17.47 ± 2.97	17.75 ± 2.75
*P* value		0.539	0.009^*∗∗*^	0.034^*∗*^	0.052

^*∗*^ *P* value indicates statistical significance (*P* < 0.05). ^*∗∗*^*P* value indicates statistical significance (*P* < 0.01).

## Data Availability

The data that support the findings of this study are available from the corresponding author upon reasonable request.
